# New Mode of Treating Perineal Fistula

**Published:** 1829-07-01

**Authors:** 


### HOPITAL DU GROS CAILLOU.

### New Mode of Treating Perineal Fistula.

It is well known to surgeons, that in the treatment of fistula in perineo the urine invariably comes away by the side of the catheter in the urethra, however large the instrument may be. Of course it must be an object to obviate, if possible, this inconvenience, and the following was the method adopted by M. Barthélemy, surgeon to the Hospital du Gros Caillou.

M. ———, an officer of Gendarmerie had been subject for many years to attacks of retention of urine for which he was in the habit of passing the catheter himself. One day he experienced more difficulty than usual, and produced a false passage into which the urine made its way, and gave rise to an urinary abscess. This broke, and a fistula in perinæo was the consequence, which after continuing open two years, closed and cicatrized. A chronic inflammation and purulent discharge from the urethra still remained, the glans penis swelled, and one day the patient discovered, very much to his surprise, that the latter part was encircled by little ulcerated openings each of which gave passage to a stream of urine. In this state he entered the Val de Grace, where injections and antiphlogistics were employed without the slightest relief. M. Barthélemy now attempted to introduce an instrument into the bladder, but it always stopped at the seat of the former fistula in perinæo. M. B. conceiving that if an instrument were passed as far as the obstruction, and retained in the urethra, the urine would flow through it, and not make its way by the ulcers at the glans, constructed a catheter, and made the experiment. The water, however, escaped by its side, and the plan not succeeding was abandoned. The idea next occurred to the surgeon of compressing the urethra around the instrument, and thus preventing the possibility of the urine insinuating itself between them. A catheter cut short, with the perforation exactly at its extremity, and not at the sides, was introduced as far as the stricture, and retained in the penis by proper tapes. The urethra was then compressed around it by a circular bandage, and the patient desired to make water. Success at length crowned the surgeon's hopes, and the joy of the comrades of Xenophon could scarcely have been greater when they burst into the tumultuous cry of "Η θαλασση θαλασση," at the right of the Euxine, than was that of M. Barthélemy, when he found, on the patient making water, that none came away by the side of the catheter! M. ———, was directed, whenever he voided his urine to apply the circular compression round the instrument, and after a month had elapsed he left the hospital perfectly cured of the fistulous ulcers of the glans.

The worst of this method is, that unless the fistula is anterior to the root of the penis, it can do no good, because, behind that point, compression on the instrument is obviously impracticable. *Journ. Univer. Janv.* 1829.

